# SeedVicious: Analysis of microRNA target and near-target sites

**DOI:** 10.1371/journal.pone.0195532

**Published:** 2018-04-17

**Authors:** Antonio Marco

**Affiliations:** School of Biological Sciences, University of Essex, Colchester, United Kingdom; John Curtin School of Medical Research, AUSTRALIA

## Abstract

Here I describe seedVicious, a versatile microRNA target site prediction software that can be easily fitted into annotation pipelines and run over custom datasets. SeedVicious finds microRNA canonical sites plus other, less efficient, target sites. Among other novel features, seedVicious can compute evolutionary gains/losses of target sites using maximum parsimony, and also detect near-target sites, which have one nucleotide different from a canonical site. Near-target sites are important to study population variation in microRNA regulation. Some analyses suggest that near-target sites may also be functional sites, although there is no conclusive evidence for that, and they may actually be target alleles segregating in a population. SeedVicious does not aim to outperform but to complement existing microRNA prediction tools. For instance, the precision of TargetScan is almost doubled (from 11% to ~20%) when we filter predictions by the distance between target sites using this program. Interestingly, two adjacent canonical target sites are more likely to be present in *bona fide* target transcripts than pairs of target sites at slightly longer distances. The software is written in Perl and runs on 64-bit Unix computers (Linux and MacOS X). Users with no computing experience can also run the program in a dedicated web-server by uploading custom data, or browse pre-computed predictions. SeedVicious and its associated web-server and database (SeedBank) are distributed under the GPL/GNU license.

## Introduction

Animal microRNAs target gene transcripts by partial sequence complementarity [1]. There are multiple microRNA target prediction tools that use different strategies. Most prediction programs look at the existence of seed sequences, six-nucleotide-long sequences in the transcripts that are complementary to nucleotides 2 to 7 in the microRNA [1]. Depending on additional nucleotide pairings these sites can be canonical or marginal. Many programs exploit additional features, mainly evolutionary conservation and RNA folding features [2,3]. Although these programs have an increased accuracy, they lose power as many real targets remain undetected. Often, microRNA target predictions are available only on selected datasets. Additionally, stand-alone programs are not always ready for use on custom data, or they are not available as web forms where users can analyse their own data without the need of specialised computing skills. Here I describe a new microRNA target prediction software that does not aim to replace but to complement the existing tool-kit, and allow the high-throughput analysis of custom microRNA/transcript data as well as the exploration of additional features not covered by other programs.

**Fig 1:**
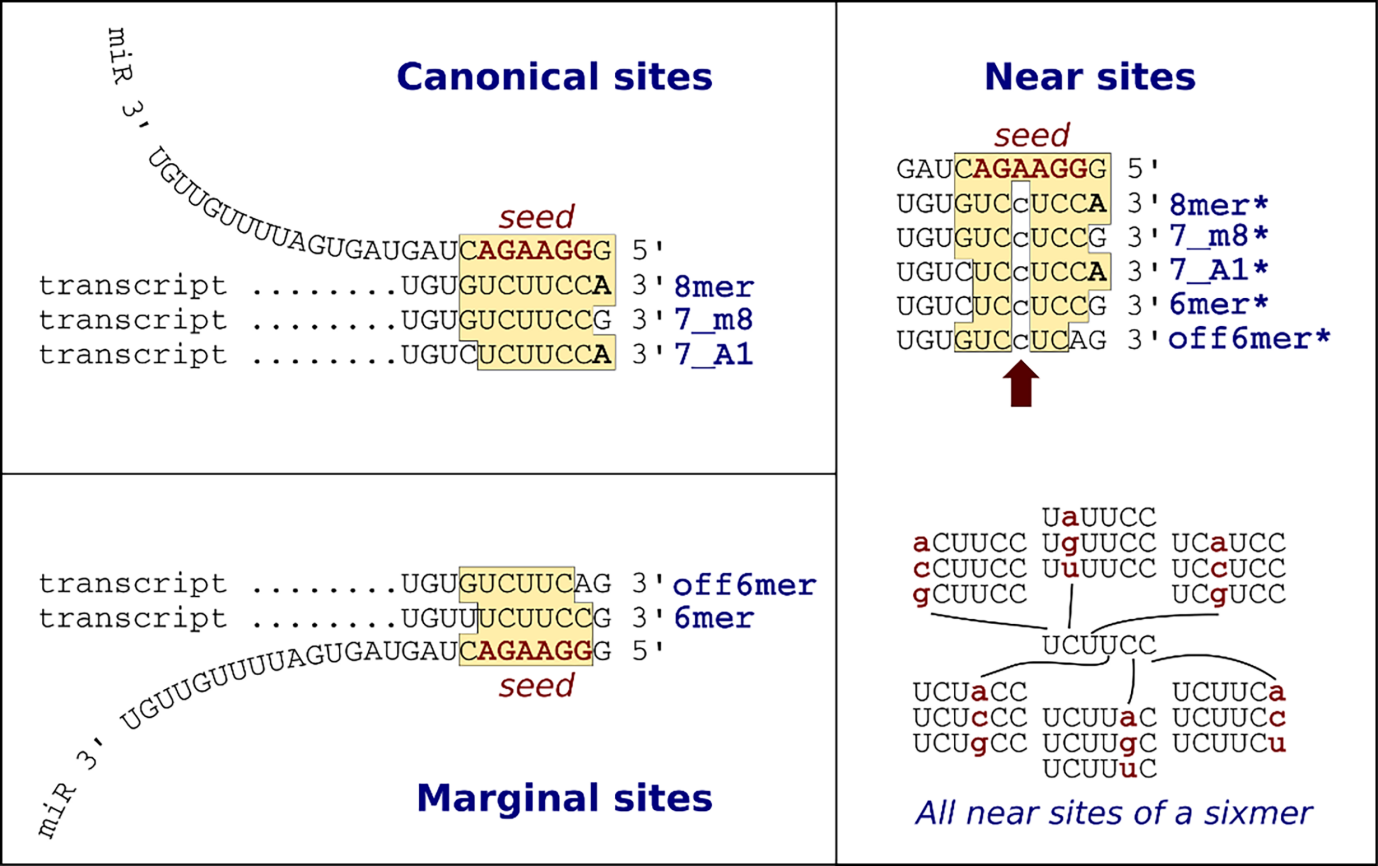
MicroRNA target and near-target sites. Canonical and marginal sites are described in Bartel, 2009 [1]. Near-target sites can be defined for all type of sites. The figure also shows that each seed (sixmer) 18 possible near-target sites as defined by Marco, 2015 [6].

The first population genetics studies on microRNA targets sites already described selective pressures on seed sequences [4,5]. In a recent study I showed that, in *Drosophila* populations, there is selection against microRNA target sites [6]. To study selection on non-target sites that may become targets, I defined ‘one-mutant neighbors’ as six-nucleotide-long sequences that have one nucleotide different to a seed sequence. Here I define a broader term: near-target sites (Fig 1), which have one nucleotide different to a putative microRNA target site, and are not targets themselves. Detection of near-target sites are important for population genetics and evolutionary studies, and will allow us to explore the selective pressures on gene transcripts. Additionally, reference genome sequences, in which microRNA target sites are usually predicted, do not capture the true diversity of a species and may miss *bona fide* target sites. Scanning for near-target sites may reveal targets that would be otherwise ignored. SeedVicious allows the exploration of near-target sites.

## Methods and implementation

SeedVicious initially scans custom sequences to detect canonical microRNA target sites as described in [1]. Optionally, marginal sites can be reported (Fig 1). This strategy produces a large number of false positives, and other prediction tools use evolutionary conservation to narrow down the number of potential target sites [2,7]. However, evolutionary conservation is not necessarily associated with functionality of the target site [8]. SeedVicious allows an alternative (and complementary) way of finding putative target sites. The complete list of canonical (and marginal) sites can be ranked according to selected features. First, seedVicious computes the free energy of the microRNA:transcript duplex using the RNAeval program in the Vienna Package [9]. The lower the energy, the more stable is the RNA:RNA pair, which can be interpreted as a more efficient target site. Second, seedVicious can also report the minimum distance between pairs of target sites for the same microRNA (the position of a target, as reported by seedVicious, is the position of the nucleotide in the transcript that is paired with the 5’ first nucleotide of the microRNA).

SeedVicious also permits the inference of gains and losses of microRNA target sites by first predicting individual target sites at all transcripts in a given alignment, and then fitting a maximum parsimony (MP) model to a tree provided by the user, following Dollo’s criteria [10]. The MP reconstruction of ancestral states is computed with the 'dollop' program from the Phylip package [11]. We first used this method to study the evolution of post-transcriptional regulation in a gene family in Drosophila [12], and as far as I’m aware, this is the only microRNA target prediction program that currently implements this type of analysis. A number of additional analyses can be performed with seedVicious. It can compare different 3’UTRs and detect either common microRNAs targeting pairs of transcripts or common target sites in an alignment. A major feature of seedVicious is the detection of near-target sites which are detected for both canonical and marginal target sites.

The main program can be run from the command line using Perl 5 or above, and the required modules and external binary files (compiled in a 64-bit Unix computer) are included in the distributed version. Input sequence files should be in FASTA format, and tree files in NEWICK format. Input files can be compressed in Gzip format. A fully referenced User Guide is available form the package or as Supplementary Information to this paper, and it includes different protocols.

For the analysis presented below, all microRNAs were retrieved from miRBase release 21 [13], and all transcript sequences from ENSEMBL Genes 89 via Biomart [14]. TargetScan targets were retrieved from targetscan.org, release 7.1 [2]. Predictions were compared to the experimentally validated targets in miRTarBase 6.0 [15], for both weak and strong interactions. Precision was calculated as the ratio TP/(TP+FP), where TP (True Positives) was the number of predicted targets that were experimentally validated, and FP (False Positives) was the number of predicted targets not validated in miRTarBase. To map near-target sites to polymorphic nucleotides the genomic coordinates of the 3’UTR of a TP53 transcript (NM_000546.5) were retrieved from UCSC Table Browser for the human genome assembly hg38 [16]. Using this information, the coordinates of the predicted near-target sites (see Results for a detailed description) were then converted into genomic coordinates. UCSC Table Browser was also used to identify all SNPs mapped to the 3’UTR of the transcript. Following this protocol, polymorphic nucleotides can be easily mapped to microRNA near-target sites.

The program is available for download at http://seedvicious.essex.ac.uk/download.html and at FigShare https://figshare.com/articles/seedVicious/6106073/1. A web version is available, which allows users to run the program using our server. Precomputed targets for selected species can also be browsed from our SeedBank database. The web server and the database are accessed via http://seedvicious.essex.ac.uk

## Results and discussion

**Fig 2:**
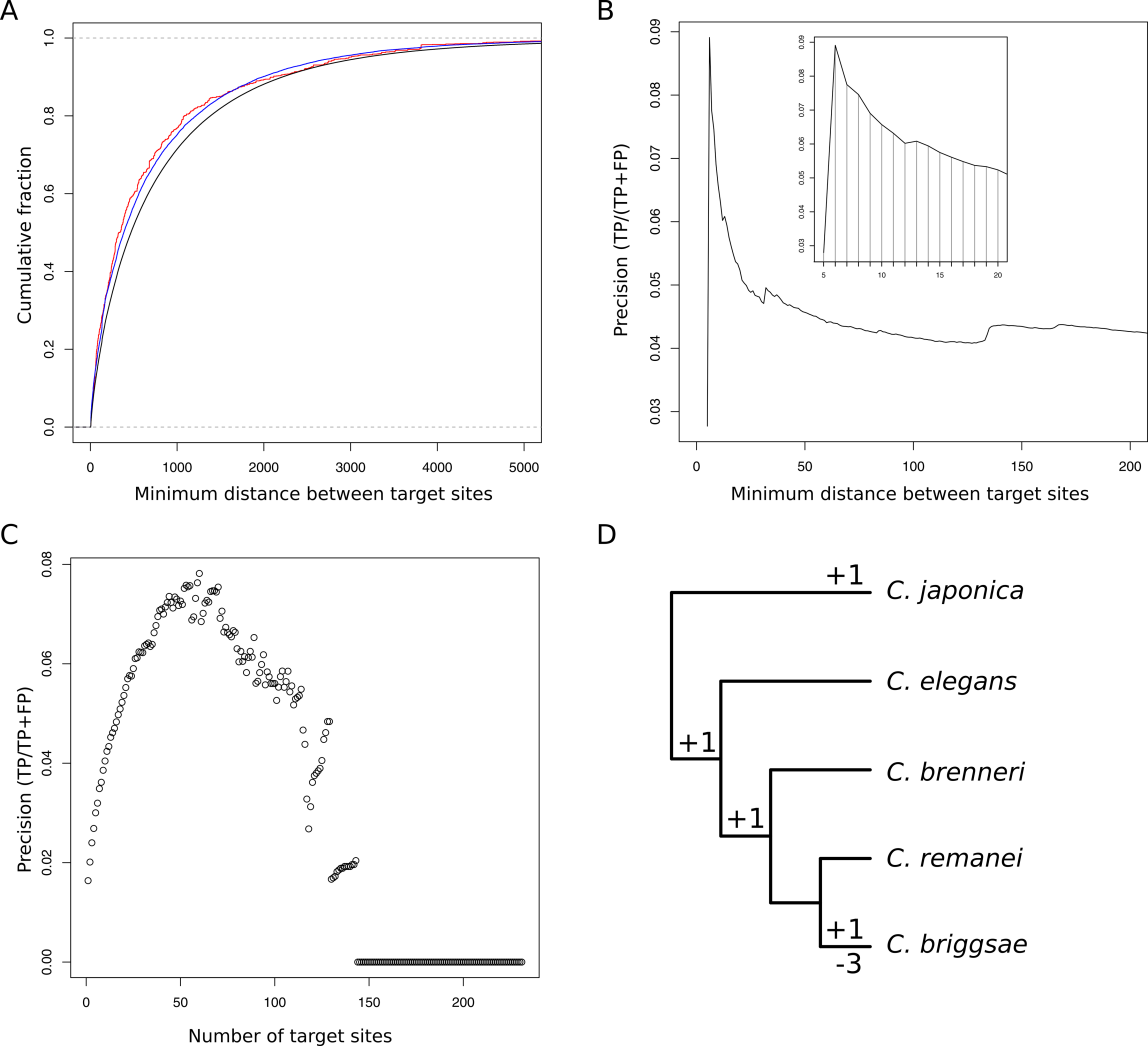
Multiple uses of seedVicious. A) Cumulative distribution of the number of transcripts with at least two target sites with respect to the minimum distance between pairs of targets. The graph compares the distribution of all transcript (black), transcript/microRNA pairs with experimentally validated interactions (blue) and those with strongly validated interactions (red) according to miRTarBase. B) Precision of microRNA target prediction for different cut-offs of minimum distance between pairs of targets. The peak corresponds to a distance of 6, that is, two contiguous target sites. C) Precision of microRNA target prediction for multiple targets sites for the same microRNA. D) Evolutionary turnover of microRNA canonical target sites for let-7 in lin-14 in roundworm species using maximum parsimony.

I describe in this section a few examples that illustrate different ways of analyzing target sites with seedVicious. First, I computed with seedVicious the minimum distance between identical canonical target sites for all human microRNAs in 3’UTRs (see Methods and Implementation). This distance can be used as a filtering criteria for potential targets, based on a recent work that suggests that neighboring sites for the same microRNA cooperate during lateral diffusion of the Ago-miRNA complex during target recognition (see [17,18] and references therein). The average minimum distance between pairs of target sites was smaller for strong (751.8) and weak (773.5) validated interactions than in all set of predicted interactions (888.1). The differences are visible when plotting the cumulative distribution of minimum distances (Fig 2A). The observed differences were statistically significant when performing a one-tailed Kolmogorov-Smirnov test between validated and predicted targets (strong versus predicted: p << 0.0001; weak versus predicted p << 0.0001). Minimum distances were also significantly smaller in strong interactions than in weak interactions (p = 0.00045). This indicates that the closest two target sites are in a transcript, the more likely that the microRNA will regulate the targeted transcript. As a matter of fact, two adjacent target sites (6 nt distance) significantly increases the precision when detecting potential target interactions (Fig 2B).

**Fig 3:**
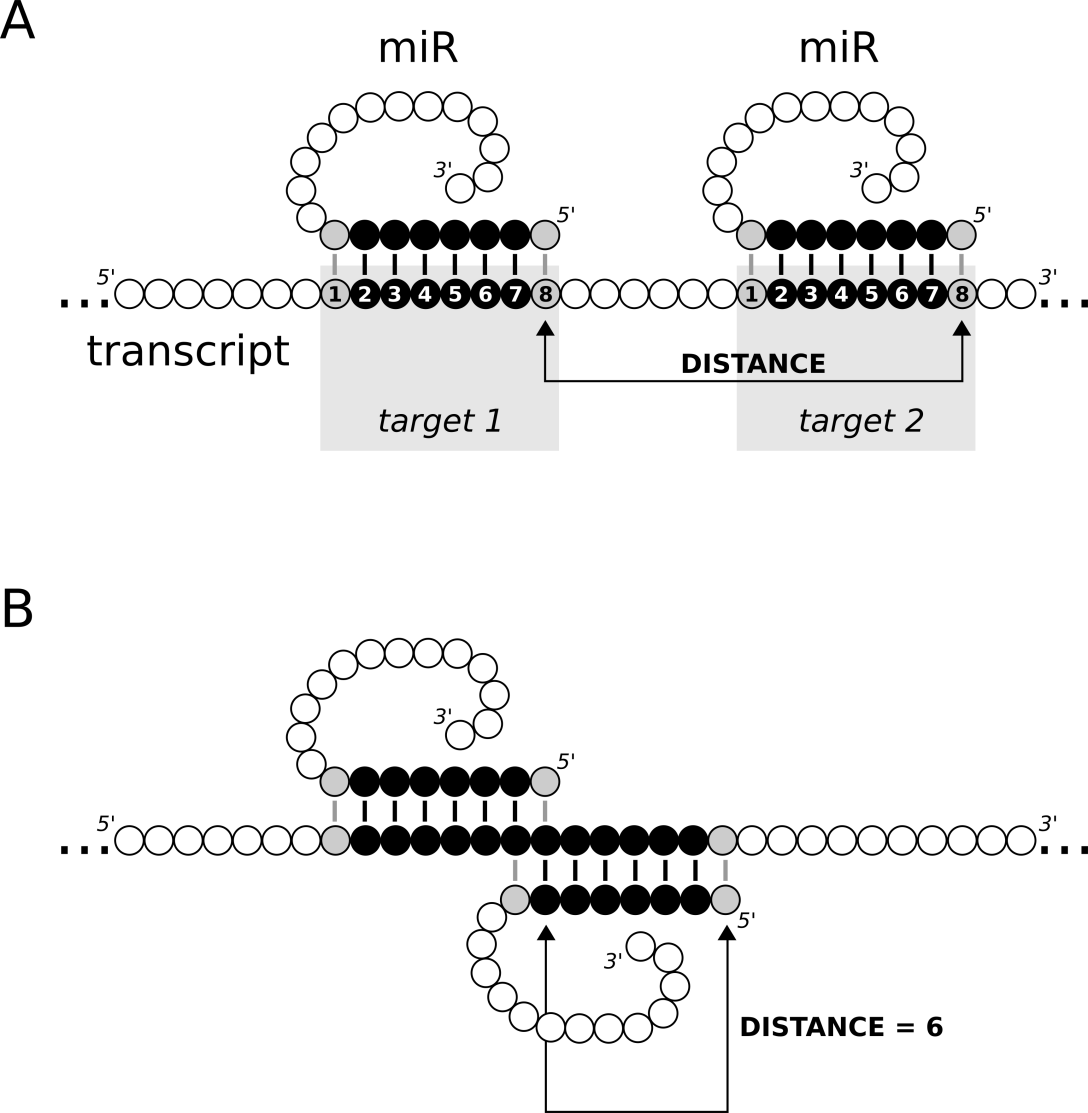
Distance between canonical target sites. Distance between canonical target sites as defined by seedVicious (A), and the partial overlap of targets that are 6 nucleotides away (B).

A distance of 6 implies that the seed sequence of two canonical target sites are adjacent, so that positions 7 and 8 of the canonical left target overlaps with the positions 1 and 2 of the right target (Fig 3). Among the microRNAs with the highest precision values at 6 nucleotides of distance we find four whose seed sequence is a dinucleotide repetitive motif. For instance, the seed sequence of miR-8485 is ACACACA so it will have multiple (and adjacent) target sites in UG-rich UTRs. Despite its low complexity, there are 371 genes predicted to be targeted by this microRNA, of which 234 interactions are also found in miRTarBase (mostly from high-throughput PAR-CLIP and HITS-CLIP experiments). That yields a markedly high precision value (63%) for this particular microRNA. Finding adjacent target sites for microRNAs with repeated dinucleotides is not surprising. However, the fact that these interactions are often found in miRTarBase is striking. An interesting possibility is that microRNA/RISC complexes bounce forth and back between clustered target sites in a one-dimensional diffusion process (see [17]), which is particularly visible for some microRNAs targeting repeated regions. In other words, multiple target sites near to each other does not imply that several microRNAs are targeting the transcript at the same time, but that a single microRNA/RISC complex may be diffusing between sites.

By using a compilation of experimentally validated microRNA/transcript interactions (see Methods and Implementation) I estimated the precision of target prediction based on the minimum distance between canonical sites. If we predict as microRNA targets transcripts with at least 2 canonical sites, the precision of the prediction is 0.041. This value significantly increases (to 0.087) if we only consider pairs of canonical sites next to each other (distance of 6 nucleotides). Precision values in microRNA target prediction are generally low, in particular when one uses a small dataset of validated targets (as in this case), but it is useful to compare the predictive power of different strategies. Using this same dataset, the precision of TargetScan (using conserved targets only) is 0.11. Strikingly, if we combine TargetScan and pairs of targets at 10 or less nucleotides in distance, the precision goes up to 0.195. In conclusion, combining some of the features of seedVicious to other programs can be use to increase the precision in microRNA target prediction.

**Fig 4:**
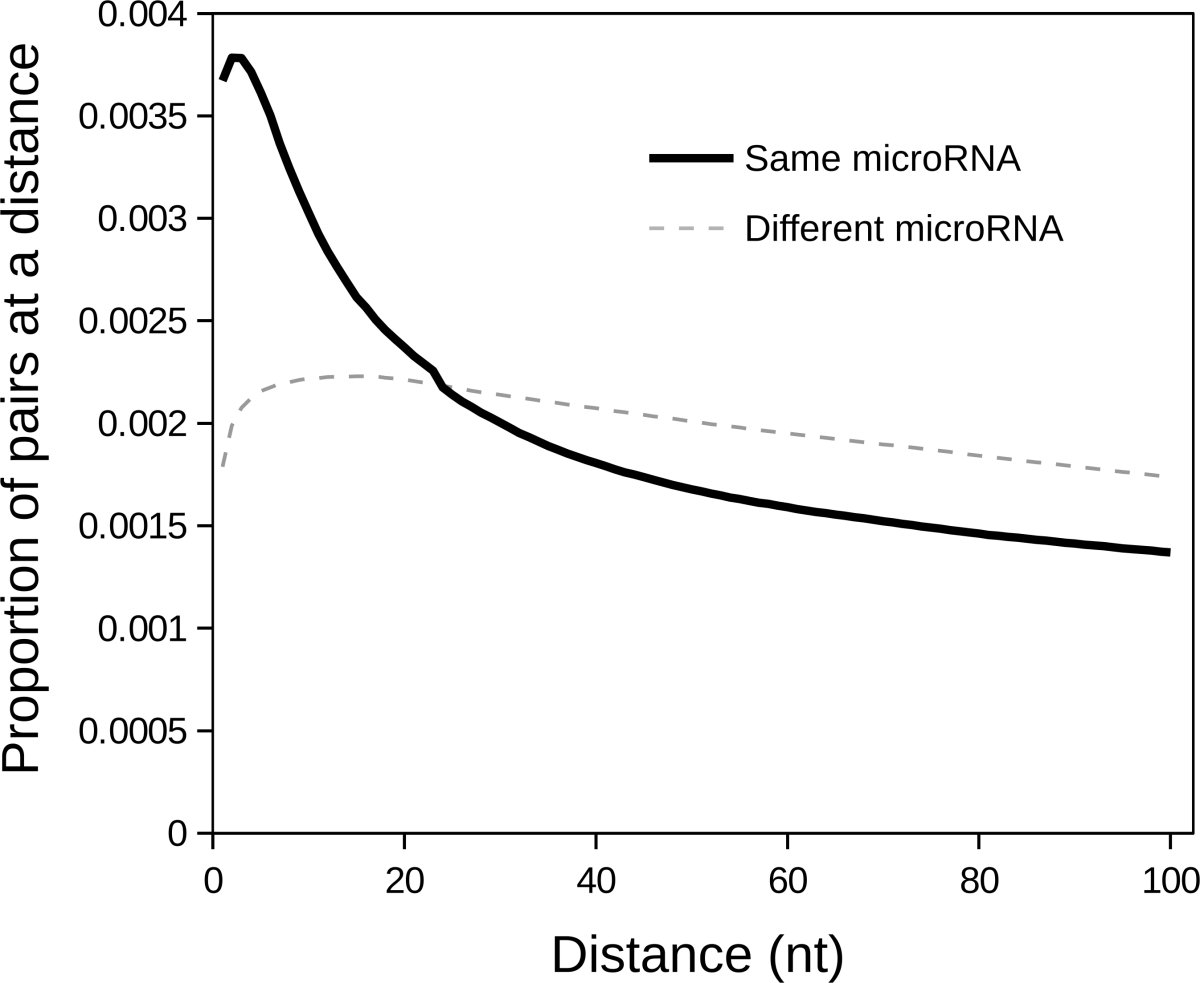
Pairs of target sites for the same and different microRNAs. Proportion of pairs of target sites (y-axis) at a given distance (x-axis) for the same microRNAs (solid black line) and for two different microRNAs (dashed gray line).

Pairs of target sites can also be for different microRNAs. Indeed, seedVicious also allows the exploration of clustered target sites for multiple microRNAs. In Fig 4 I plot the proportion of pairs of targets at a given distance for all pairs of microRNAs in the human genome. For distances of up to about 20 nucleotides, pairs of target sites are more likely to be for the same microRNA than to different microRNAs. However, this pattern is probably a consequence of sequence bias composition: if a segment of a 3’UTR is rich in a given motif, it is more likely that microRNAs with the same seed sequence (same family) will target at nearby places. For instance, RNA-binding proteins (RBPs) often bind to clustered motifs (see [19] and references within).

In previous studies, the occurrence of two or more seed sequences in transcripts has been associated to a higher chance of predicting a *bona fide* targeting interaction [20]. Here I compared the proportion of predicted targets that are validated by experiments (precision) and the number of canonical target sites. The graph in Fig 2C shows that, indeed, as the number of target sites increase, the proportion of validated targets also increases, although there is a global maximum around 50. It will be interesting to explore whether distinct microRNAs have a different optimum number of target sites for an efficient repression of the target transcript.

The analysis of targets and near-targets is of particular interest to analyse populations with segregating alleles in target sites [4,6]. For instance, it has been shown that amplifications of the microRNAs mir-17, mir-18 and mir-19 from the *mir-17~mir-92* cluster lead to tumor development (reviewed in [21]). That is, this cluster is an oncogenic loci. I scanned the 3’UTR sequence of *TP53* with seedVicious to find near-target sites of the oncogenic microRNAs of the cluster and I found 14 sites. Comparing these sites with polymorphic nucleotides (see Methods) I detected one specific target site for miR-18a-5p (dbSNP accession number rs17881366P) that it is a non-target in the reference genome, but a small yet significant fraction of the human population (0.6%) has a target allele. In populations of African ancestry the target allele is detected in over 2% of the individuals sequenced. Whether a target site for a oncogenic microRNA in a tumour-suppresor gene segregation in African populations have a significance from the epidemiological point of view cannot be inferred, and this result remains largely speculative. However, it demonstrates that this methodology can be adapted to explore the impact of microRNA target sites segregating in human populations. Indeed, current work in our lab reveals that selective pressures against this type of substitutions in cancer-associated genes is prevalent (Hatlen and Marco, unpublished results).

The transcript from the gene *lin-14* in *Caenorhabditis elegans* has seven targets sites for the microRNA *lin-4* [22]. However, other prediction programs detect only three (TargetScan) or none, probably due to the stringent criteria. Using seedVicious I scanned the lin-14 3’ UTR [23] for canonical target and canonical near-target sites. Three canonical sites were detected, the same that are reported in TargetScan. Additionally, five near-target sites were reported, four of them corresponding to the other four sites originally described. One of the near-target sites was not previously described and may be a good candidate to further explore. The detailed analysis as well as the input files are available with the package and fully described in the User Guide (S1 File). This example illustrates the potential of studying near-target sites, not only in evolutionary studies, but in the detection of potential functional targets. However, these near-target sites may actually be target sites segregating in a population. The exploration of segregating microRNA target sites in populations is a promising area of research. Further examples are described in the software manual.

The canonical target sites for lin-4-5p in lin-14 are highly conserved. Actually, in five species of worm studies all sites remain the same. However, let-7-5p, which also targets lin-14, has a more dynamic evolution. Using seedVicious I predicted the canonical target sites in five worm species and to reconstruct the gains and losses using maximum parsimony. Results are shown in Fig 2D, where we observe that there have been 4 target sites gained among the studied species whilst *Caenorhabditis briggsae* lost its three ancestral target sites.

## Conclusion

SeedVicious is a valuable addition to the toolkit of microRNA biologists. It works on custom datasets and can be easily fitted into annotation pipelines. It provides a range of analysis that can be combined with other programs to generate robust microRNA target predictions. The analysis of near-target sites is also useful to study population dynamics at target sites, and the inbuilt maximum parsimony functionality is valuable to study the evolution of microRNA-mediated regulation at a large scale.

## Supporting information

S1 File User guide and installation instructions for seedVicious software. It includes protocols and recipes.
(PDF)

## References

[1] Bartel DP. MicroRNAs: target recognition and regulatory functions. Cell. 2009;136: 215–233. doi: 10.1016/j.cell.2009.01.002 19167326 10.1016/j.cell.2009.01.002 PMC3794896

[2] Agarwal V, Bell GW, Nam J-W, Bartel DP. Predicting effective microRNA target sites in mammalian mRNAs. eLife. 2015;4 doi: 10.7554/eLife.05005 26267216 10.7554/eLife.05005 PMC4532895

[3] Maragkakis M, Alexiou P, Papadopoulos G, Reczko M, Dalamagas T, Giannopoulos G, et al Accurate microRNA target prediction correlates with protein repression levels. BMC Bioinformatics. 2009;10: 295 doi: 10.1186/1471-2105-10-295 19765283 10.1186/1471-2105-10-295 PMC2752464

[4] Chen K, Rajewsky N. Natural selection on human microRNA binding sites inferred from SNP data. Nat Genet. 2006;38: 1452–1456. doi: 10.1038/ng1910 17072316 10.1038/ng1910

[5] Saunders MA, Liang H, Li W-H. Human polymorphism at microRNAs and microRNA target sites. Proc Natl Acad Sci U S A. 2007;104: 3300–3305. doi: 10.1073/pnas.0611347104 17360642 10.1073/pnas.0611347104 PMC1805605

[6] Marco A. Selection Against Maternal microRNA Target Sites in Maternal Transcripts. G3 GenesGenomesGenetics. 2015;5: 2199–2207. doi: 10.1534/g3.115.019497 26306531 10.1534/g3.115.019497 PMC4593001

[7] Paraskevopoulou MD, Georgakilas G, Kostoulas N, Vlachos IS, Vergoulis T, Reczko M, et al DIANA-microT web server v5.0: service integration into miRNA functional analysis workflows. Nucleic Acids Res. 2013;41: W169–W173. doi: 10.1093/nar/gkt393 23680784 10.1093/nar/gkt393 PMC3692048

[8] Pinzón N, Li B, Martinez L, Sergeeva A, Presumey J, Apparailly F, et al The number of biologically relevant microRNA targets has been largely overestimated. Genome Res. 2016;

[9] Hofacker IL. RNA secondary structure analysis using the Vienna RNA package. Curr Protoc Bioinforma Ed Board Andreas Baxevanis Al. 2009;Chapter 12: Unit12.2. doi: 10.1002/0471250953.bi1202s26 19496057 10.1002/0471250953.bi1202s26

[10] Felsenstein J. Inferring Phylogenies. Sinauer Associates; 2003.

[11] Felsenstein J. PHYLIP (Phylogeny Inference Package) version 3.6 Distributed by the author. Department of Genome Sciences, University of Washington, Seattle; 2005.

[12] Clifton BD, Librado P, Yeh S-D, Solares ES, Real DA, Jayasekera SU, et al Rapid Functional and Sequence Differentiation of a Tandemly Repeated Species-Specific Multigene Family in Drosophila. Mol Biol Evol. 2017;34: 51–65. doi: 10.1093/molbev/msw212 27702774 10.1093/molbev/msw212 PMC6404660

[13] Kozomara A, Griffiths-Jones S. miRBase: annotating high confidence microRNAs using deep sequencing data. Nucleic Acids Res. 2014;42: D68–73. doi: 10.1093/nar/gkt1181 24275495 10.1093/nar/gkt1181 PMC3965103

[14] Kinsella RJ, Kähäri A, Haider S, Zamora J, Proctor G, Spudich G, et al Ensembl BioMarts: a hub for data retrieval across taxonomic space. Database J Biol Databases Curation. 2011;2011: bar030 doi: 10.1093/database/bar030 21785142 10.1093/database/bar030 PMC3170168

[15] Chou C-H, Chang N-W, Shrestha S, Hsu S-D, Lin Y-L, Lee W-H, et al miRTarBase 2016: updates to the experimentally validated miRNA-target interactions database. Nucleic Acids Res. 2016;44: D239–D247. doi: 10.1093/nar/gkv1258 26590260 10.1093/nar/gkv1258 PMC4702890

[16] Karolchik D, Hinrichs AS, Furey TS, Roskin KM, Sugnet CW, Haussler D, et al The UCSC Table Browser data retrieval tool. Nucleic Acids Res. 2004;32: D493–496. doi: 10.1093/nar/gkh103 14681465 10.1093/nar/gkh103 PMC308837

[17] Chandradoss SD, Schirle NT, Szczepaniak M, MacRae IJ, Joo C. A Dynamic Search Process Underlies MicroRNA Targeting. Cell. 2015;162: 96–107. doi: 10.1016/j.cell.2015.06.032 26140593 10.1016/j.cell.2015.06.032 PMC4768356

[18] Denzler R, McGeary SE, Title AC, Agarwal V, Bartel DP, Stoffel M. Impact of MicroRNA Levels, Target-Site Complementarity, and Cooperativity on Competing Endogenous RNA-Regulated Gene Expression. Mol Cell. 2016;64: 565–579. doi: 10.1016/j.molcel.2016.09.027 27871486 10.1016/j.molcel.2016.09.027 PMC5101187

[19] Zhang C, Lee K-Y, Swanson MS, Darnell RB. Prediction of clustered RNA-binding protein motif sites in the mammalian genome. Nucleic Acids Res. 2013;41: 6793–6807. doi: 10.1093/nar/gkt421 23685613 10.1093/nar/gkt421 PMC3737533

[20] Alexiou P, Maragkakis M, Papadopoulos GL, Reczko M, Hatzigeorgiou AG. Lost in translation: an assessment and perspective for computational microRNA target identification. Bioinformatics. 2009;25: 3049–3055. doi: 10.1093/bioinformatics/btp565 19789267 10.1093/bioinformatics/btp565

[21] Concepcion CP, Bonetti C, Ventura A. The miR-17-92 family of microRNA clusters in development and disease. Cancer J Sudbury Mass. 2012;18: 262–267. doi: 10.1097/PPO.0b013e318258b60a 22647363 10.1097/PPO.0b013e318258b60a PMC3592780

[22] He L, Hannon GJ. MicroRNAs: small RNAs with a big role in gene regulation. Nat Rev Genet. 2004;5: 522–531. doi: 10.1038/nrg1379 15211354 10.1038/nrg1379

[23] Jan CH, Friedman RC, Ruby JG, Bartel DP. Formation, regulation and evolution of Caenorhabditis elegans 3[prime]UTRs. Nature. 2010;advance online publication. doi: 10.1038/nature09616 21085120 10.1038/nature09616 PMC3057491

