# Supplementary material for: SeedVicious: Analysis of microRNA target and near-target sites
Source: PLoS One. 2018 Apr 17;13(4):e0195532. doi: 10.1371/journal.pone.0195532 (PMC5903666; doi:10.1371/journal.pone.0195532)
Supplement: S1 File — It includes protocols and recipes. (PDF) [file pone.0195532.s001.pdf]

seedVicious 1.2

User Guide

Antonio Marco

University of Essex

School of Biological Sciences

©2017 Antonio Marco

http://amarco.net/

This user guide is licensed under a Creative Commons
Attribution-ShareAlike 4.0 International License:

http://creativecommons.org/licenses/by-sa/4.0/

User Guide version 1.2 (5th March 2018)

The software described, seedVicious, is distributed under the GNU GENERAL PUBLIC LICENSE v3.0 (see LICENSE file distributed with the package). Software is provided without warranty. This license does not apply to the external programs included with this package: dollop is part of the Phylip package, distributed with an open source license, and available at: http://evolution.genetics.washington.edu/phylip.html; RNAeval is part of the ViennaRNAPackage, distributed as free software, and available at: https://www.tbi.univie.ac.at/RNA/

Cover image designed by Antonio Marco.

Preface

This manual describes the basic functions of seedVicious, a microRNA target site prediction program. It will also provide some protocols exploring the different functions. The text is organized as follows. Chapter 1 gives a brief introduction to microRNA target prediction. Chapter 2 describes the installation of seedVicious and the input files accepted. Chapter 3 lists the different options of the program and how they work. Chapter 4 contains some useful protocols that can be adapted to the user's needs. Last chapter includes frequently asked questions and some useful information.

Although seedVicious is a personal project (a kind of a one-man show) it has been benefited by the feedback and comments of many colleagues, including Sam Griffiths-Jones, Matt Ronshaugen, Maria Ninova, Mohab Helmy, Andrea Hatlen, Roman Cheplyaka, Stuart Newman and a number of anonymous and less anonymous reviewers, among others. However, any error or bug is my fault (but remember that the software comes with no warranty).

For any suggestion or question write me at `amarco.bio@gmail.com`.

Colchester, United Kingdom
April 2017 — Antonio Marco
(last updated in Mach 2018)

Contents

1 MicroRNA target sites **3**

1.1 What's a microRNA 3

1.2 Detection of MicroRNA target sites 4

1.3 Why seedVicious? 5

2 Setting-up seedVicious **7**

2.1 Requirements 7

2.2 Download, requirements and installation 8

2.3 Input files 8

3 Predicting target sites with seedVicious **11**

3.1 Basic detection of MicroRNA target sites 11

3.2 File parsing options 13

3.3 Additional analysis: overview 14

3.3.1 `-x 1`: Common targets 14

3.3.2 `-x 2`: Common target sites 15

3.3.3 `-x 3`: Ancestral state reconstruction 15

3.3.4 `-x 4`: Pairs of targets 16

3.4 Web-server version 16

3.5 SeedBank database of pre-computed targets 17

4 Protocols 19

4.1 Detection of targets and near target between lin-4 and lin-14 . 19

4.2 Gains/losses of let-7 targets in lin-14 21

4.3 Exploring lin-4 and let-7 regulated transcripts 23

4.4 Exploring alternative splicing and microRNA targets 26

4.5 Clusters of microRNA targets 26

5 Frequently Asqued Questions 29

Bibliography 31

1

MicroRNA target sites

If you are reading this manual, you probably know what a microRNA is, and how it binds to transcripts. In any case, I included in this chapter a very brief introduction to microRNAs, and some relevant information regarding target prediction algorithms. Please note that seedVicious is a program to analyze animal microRNA target sites and, tehrefore, this introductory chapter will focus only on animal microRNAs.

1.1 What's a microRNA

The textbook definition of a microRNA is that a microRNA is a short endogenous RNA molecule, about 21 nucleotides long, that represses protein translation by targeting transcripts, mostly by binding to their 3' untranslated regions (UTR) by partial complementarity [4, 3]. When the first microRNA was discovered in *Caenorhabditis elegans* (the roundworm) it was already found that the short RNA molecule produced by its gene (*lin-4*) had multiple partially-complementary sites on the 3' UTR of the transcripts produced by its putative gene target, lin-14 [38, 23]. But this sort of small molecules were not found in other species. A second small RNA coding genes was discovered in *C. elegans* a few years later, *let-7* [35]. This time, homologs of *let-7* were found in other species, including human [33]. Soon after, dozens of small-RNA encoding genes were described in humans, flies, mice and the roundworm [22, 21, 19] , and the term microRNA was coined. Now we know

thousands of microRNAs in pretty much all studied animals [16], and also in plants and some unicellular organisms, but these are not covered here.

A few more interesting facts about microRNAs: They are often encoded in operons, that is, the same transcripts may have several microRNA sequences, and can also be within the introns of protein-coding genes (reviewed in [30]). Potentially, a single microRNA locus produces two mature sequences from a precursor hairpin. Pairs of microRNAs from the same precursor target different sets of genes [29].

1.2 Detection of MicroRNA target sites

The fact that microRNAs bind to target transcripts by partial complementarity is very convenient from the computational point of view. In principle, by finding the rules by which partial complementarity leads to translation repression, we can easily predict binding sites. The are tens of early studies that dissected the properties of microRNA/transcripts binding (see [10, 20, 25, 24, 6, 37, 12]). A good review on the topic is still that of David Bartel in 2009 [5] (see also [1]). The problem is that it is not that easy to predict sites by using only pairwise complementarity and, therefore, different prediction programs use different additional strategies to refine their searches.

Many programs start their searches by finding canonical sites (as defined in [5]), see Figure 1.1. Then they take into account additional features such as evolutionary conservation of target sites, or the free energy of the formed RNA duplex. Table 1.1 is a non-exhaustive list of microRNA target prediction programs and their main features. A very interesting and recommended review of different tools is that of Artemis Hatzigeorgiou and collaborators in 2009 [2].

The goal of microRNA target sites prediction tools is to come up with a list of potential targets, that later on will be experimentally validated. Therefore, a stringent criteria is often applied. We may loss power and many *bona fide* targets will no be reported. But we will gain accuracy, and the proportion of false positives will be kept reasonable small.

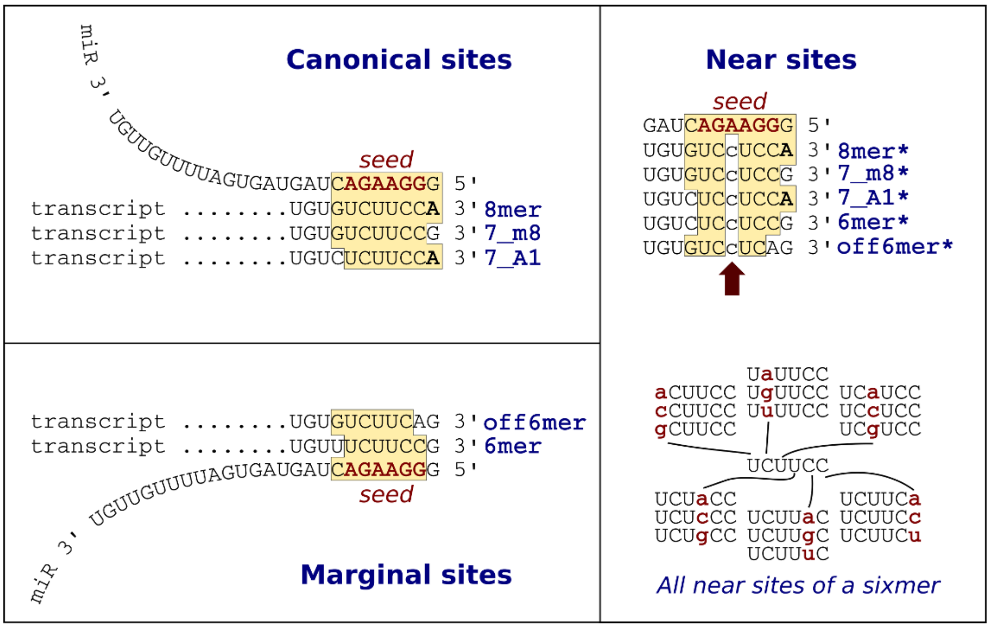

Figure 1.1: Canonical and marginal MicroRNA target sites (left). Near-target sites (right)

1.3 Why seedVicious?

First of all I must say that seedVicious does not aim to replace any existing microRNA prediction software. Each program has its own strengths and weaknesses. During my research I encountered some difficulties which I solved by developing my own target prediction program. An early version of this program dates back to 2009 and has been used by me or other colleagues [29, 28, 26, 31, 17]. However, it was never a stand-alone program until now. But, why it's worth making it publicly available? Well, here are some of the features that I think can be of use.

In high-throughput analyses it is often convenient to scan transcripts for canonical seeds without any additional filtering step like conservation. This is particularly useful when studying evolution, to avoid the circular reasoning of using evolutionary conservation to infer evolutionary conservation. That's how seedVicious started. But as my research developed into the population dynamics of microRNA targets I realized that there where no programs that will scan for near-target sites (see Figure 1.1). This feature is indeed useful

**Software**	**Ref.**	**Features**
seedVicious	[27]	Described in this User Guide.
TargetScan	[1]	Popular program. Finds canonical and marginal sites and uses evolutionary conservation. Stand-alone version available but not well documented. Web-server does not allow the exploration of custom datasets.
miRanda	[10]	Mostly based on the thermodynamic properties of the microRNA/transcripts binding. Allows wobble pairs. Runs on custom datasets.
Diana-microT	[32]	One of the most accurate target prediction programs. It includes multiple features (like conservation and binding energy). Prediction only available for pre-computed datasets.
PicTar	[8]	Based on canonical seeds, allowing wobble pairs. Available for pre-computed datasets.
RNAhybrid	[18]	Predictions are based on their thermodynamic properties. Runs on custom datasets.

Table 1.1: MicroRNA target prediction programs

to study the selective pressures on target sites [26]. Actually, my current research programme, funded by the Wellcome Trust (WT), exploits this feature. It is thanks to the WT that I could put some time into the building of the stant-alone version, putting together pre-existing scripts, and developing the web-server and the full near-target sites function.

Other additional functions have been developed through the years, and I included them in seedVicious. For instance, the use of maximum parsimony to predict the gains/losses of microRNA target sites during evolution [9]. Or the distance between pairs of canonical microRNA target sites. These and other features are described in this User Guide, and some practical protocols are provided.

In summary, seedVicious is not (nor it aims to be) the best of microRNA target prediction programs. But it offers an additional set of tools and a convenient web-server that will help researchers to make the most of their data. I hope you find it useful.

2

Setting-up seedVicious

Here I introduce seedVicious, a microRNA target site prediction program that detects canonical sites plus other additional features. seedVicious is written in Perl 5, and at the heart of it is the module `Mitargets.pm` which can be used in other Perl scripts. A detailed documentation of this class is not yet available. Some features use external programs that are distributed together with this package. However, at the moment I only provide 64-bit compiled versions for Linux and Mac OS X. I aim to provide a Microsoft Windows version soon, as well as 32-bit compiles, but at the moment it is not a priority. Give a shout if you need those and I'll do my best.

All commands in this user guide are written for a Bash/Unix environment.

2.1 Requirements

SeedVicious works on a UNIX environment (GNU/Linux or MacOS X). You will need to have Perl 5 or above installed in your system. If you want to read gzip compressed files, seedVicious will require the PerlIO::gzip module. `http://search.cpan.org/~nwclark/PerlIO-gzip-0.19/gzip.pm`

You can install it from CPAN using the command-line. You will need root privileges to install it:

```
sudo cpan
```

```
install PerlIO::gzip
```

2.2 Download, requirements and installation

Download seedVicious using `curl`:

```
curl "http://seedvicious.essex.ac.uk/seedVicious_v1.1_x64.tar.gz"
      -o "seedVicious_v1.1_x64.tar.gz"
```

Uncompress the package:

```
tar -xvf seedVicious_v1.1_x64.tar.gz
```

Move the folder to your preferred location (e.g.)

```
mv seedVicious_v1.1_x64 ~/software
```

Edit the /.bashrc file to add the folder to the PATH:

```
echo 'export PATH=$PATH:~/software/seedVicious_v1.1_x64'
      >> ~/.bash_profile
```

Reload the PATH:

```
source ~/.bash_profile
```

Test the program:

```
seedViciousTest
```

If no error is produced, you can start using seedVicious. The tag `--help` describe the basic usage:

```
seedVicious --help
```

2.3 Input files

Sequence input files must be in FASTA format. For instance, the file `cel-let-7-5p.fas` in the `datasets` folder looks like this:

```
>cel-let-7-5p
UGAGGUAGUAGGUUGUAUAGUU
```

Although microRNA target prediction requires only RNA sequences, if DNA sequences are provided there will be automatically formatted as RNA ('U' instead of 'T').

A FASTA file may contain multiple sequences. If multiple transcripts are provided, and several transcripts derive from the same gene, I recommend you use the naming suggested by ENSEMBL (`http://www.ensembl.org/`) and write the name of the gene followed by the name of the transcript, separated by a vertical bar (|). For instance:

```
>GeneA|TranscriptA1
ATTTCAATCA
>GeneA|TranscriptA2
ATTTCAATCACAAATGCCTTTTTTAAAACCAAACTAAA
>GeneA|TranscriptA3
ATTTCAATCACAAATGCCTTTTTTGGAGAGAATTGAAGGCAAAACCAAACTAAA
>GeneB|TranscriptB1
CCGTTTTAGCTTTTAATGTTAAAATCAGGAACTTTTGAA
>GeneB|TranscriptB2
CCGTTTTAGCTTTTAATGTTA
```

seedVicious can then select the longest 3'UTR for each gene (if the option is activated, as I describe below).

For comparative analysis you should provided aligned sequences, also in FASTA format. For evolutionary analysis you will need a phylogenetic tree of the transcript sequences in the input file. This tree must be in rooted Newick format (`http://evolution.genetics.washington.edu/phylip/newicktree.html`).

For instance, the file `lin-14_3UTR.tre` in the `datasets` folder is a Newick tree of 5 worm species whose transcripts for the gene *lin-14* will be analysed a later chapter:

```
((((C._briggsae,C._remanei),C._brenneri),C._elegans),C._japonica);
```

This tree is the Newick representation of the phylogenetic tree shown in Figure 2.1

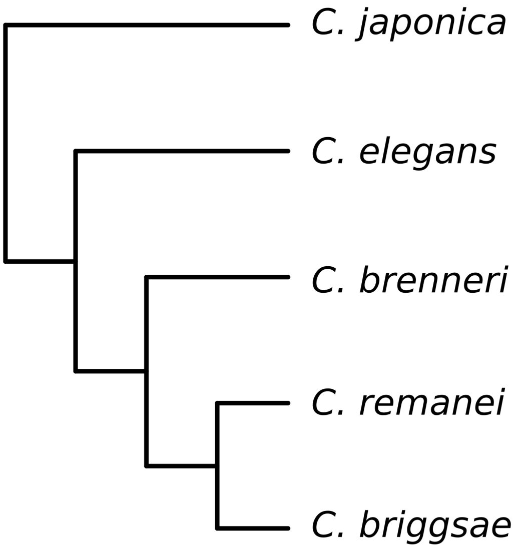

Figure 2.1: Phylogenetic relationships between five species of the *Caenorhabditis* genus

3

Predicting target sites with seedVicious

3.1 Basic detection of MicroRNA target sites

Here I briefly summary the different options that seedVicious has, and the format of the output files. Practical examples will be described in the next chapter.

The basic microRNA target prediction requires only an input transcript file (`-i`) and an input microRNA file (`-m`):

```
seedVicious -i input_transcripts.fas -m input_microRNAs.fas
```

(Make sure the microRNA input file contains only the mature sequences and not the precursors. It may look obvious, but I've seen it in the past.)

The output will be directed to the standard output, and will look like this:

```
miR         tr          pos         type
cel-let-7-5p    cel-lin-14      356     8mer
cel-let-7-5p    cel-lin-14      809     8mer
cel-let-7-5p    cel-lin-14      823     8mer
cel-let-7-5p    cel-lin-14      1410    7_A1
```

Where the third column is the position of the last nucleotide of the target

site in the transcript, and the last column is the type of target, as described in Bartel, 2009 [5] (see Figure 1.1). The output can be redirected to a file using the tag `-o`:

```
seedVicious -i input_transcripts.fas -m input_microRNAs.fas
            -o output_file.txt
```

If you want to additionally compute the hybridization energy of the microRNA/target interaction (using the `RNAeval` program from the Vienna RNA Package [14]), you can add the tag `-e` to the program call:

```
seedVicious -i input_transcripts.fas -m input_microRNAs.fas -e
```

So the output will be:

```
miR       tr        pos       type      en
cel-let-7-5p    cel-lin-14      356       8mer      -4.54 Kcal/mol
cel-let-7-5p    cel-lin-14      809       8mer      -9.10 Kcal/mol
cel-let-7-5p    cel-lin-14      823       8mer      -7.39 Kcal/mol
cel-let-7-5p    cel-lin-14      1410      7_A1      -5.30 Kcal/mol
```

For parsing purposes, it is often convenient to not to print the header of the output table. For this, you just add the tag `-c` to the command. Also, you can get a more detailed output including an alignment of the microRNA and the target site, using the tag `-v` ('verbose' mode):

```
seedVicious -i input_transcripts.fas -m input_microRNAs.fas -ve
```

(Note that we can combine several options together. In this case, the `-e` and `-v` can be combined into a single tag `-ev`.)

The output will look like this:

```
>cel-let-7-5p@cel-lin-14:356
MicroRNA   = cel-let-7-5p
Transcript = cel-lin-14
Position   = 356
Type       = 8mer

miR  3' UUGAUAUGUUGGAUGAUGGAGU 5'
        |       |   | ||||||||
tr   5' AUUAUGCAACAAUUCUACCUCA 3'
```

```
>cel-let-7-5p@cel-lin-14:809
MicroRNA   = cel-let-7-5p
Transcript = cel-lin-14
Position   = 809
Type       = 8mer

miR  3' UUGAUAUGUUGGAUGAUGGAGU 5'
          |   |    || ||||||||
tr   5' CUCAGGAAUUUCUUCUACCUCA 3'

[...]
```

The program also detects marginal sites (Figure 1.1). To report sixmers add the tag `-6`. To report offsixmers add the tag `-9`. As in the previous case you can combine both tags in one to report both types of marginal sites:

```
seedVicious -i input_transcripts.fas -m input_microRNAs.fas -69
```

Last but not leat, seedVicious will find near-target sites (as described in the previous chapter and Figure 1.1). This is done with the tag `-n`. Near-target sites are marked with an asterisk in the output:

```
miR      tr       pos      type
cel-let-7-5p    cel-lin-14      356      8mer
cel-let-7-5p    cel-lin-14      809      8mer
cel-let-7-5p    cel-lin-14      823      8mer
cel-let-7-5p    cel-lin-14      1222     8mer
cel-let-7-5p    cel-lin-14      1410     7_A1
cel-let-7-5p    cel-lin-14      584      7_A1*
cel-let-7-5p    cel-lin-14      958      7_A1*
cel-let-7-5p    cel-lin-14      1190     7_m8*
cel-let-7-5p    cel-lin-14      419      7_m8*
```

3.2 File parsing options

Input files can be parsed before the target prediction. MicroRNAs with the same 'extended' seed sequence (nucleotides 1 to 8) will have the same targets (as defined in Figure 1.1). Thus, you can merge all these microRNAs into

so-called microRNA seed families[1] using the optional tag `-f`. Note that if this option is selected, it will not be possible to compute hybridization energies.

Likewise, when multiple transcripts exists for the same gene one can select only the longest isoform. Indeed, you may be missing alternative isoforms that are biologically relevant, but as a first approximation when working with large datasets, it is very useful. You can do this with the tag `-l`.

By default, seedVicious assume that the input transcript sequences are not aligned. If you are working with alignment and want to know the position of the targets in both, the sequence and the alignment, you should use the tag `-a`.

I also added an extra feature that I found useful in the past, when exploring non-annotated regions of the genome. By adding the option `-r 2` seedVicious will scan both the forward and the reverse strand of the input transcript file. With `-r 1` it will explore only the reverse strand.

3.3 Additional analysis: overview

Apart from the target prediction, there are 4 additional analyses implemented in seedVicious. These are called with the tag `-x` followed by a number: 1 for common targets; 2 for common target sites; 3 for ancestral state reconstruction; and 4 for pairs of targets. By calling one of these analysis the program will report both the target prediction and the additional analysis. If you only require the additional analysis you can specify this tot he program by writing twice the number (For instance `-x 33`).

3.3.1 `-x 1`: Common targets

Let's suppose you want to know the number of common microRNAs targeting a pair of transcripts. With seedVicious you can compute this relatively easy:

```
seedVicious -i input_transcripts.fas -m input_microRNAs.fas -x 1
```

[1]Not to be confused with the term 'MicroRNA families' which refers to evolutionarily related microRNA genes

For instance, for three transcripts the output will look like this:

```
transcript       transcript       common_miRs
transcript_A     transcript_B        4            [miR-4918-5p...
transcript_B     transcript_C        2            [miR-4918-5p...
transcript_A     transcript_C        2            [miR-3643-5p...
```

Where the third column is the number of common microRNAs in their targets and the fourth (unnamed) column is the list of those microRNAs.

3.3.2 -x 2: Common target sites

Similar to the previous option, we can compute the number of common microRNA target sites. For that, the input transcript file should be an alignment:

```
seedVicious -i input_aligned_transcripts.fas -m input_microRNAs.fas -x 2
```

The output will include the position in the alignemnt of each of the reported target sites:

```
transcript       transcript       common_miRs
transcript_A     transcript_B        5            [miR-4918-5p>159...
transcript_B     transcript_C        3            [miR-4918-5p>159...
transcript_A     transcript_C        3            [miR-3643-5p>201...
```

3.3.3 -x 3: Ancestral state reconstruction

The gains and losses of microRNA target sites can be reconstructed using a Maximum Parsimony (MP) algorithm. seedVicious infer ancestral states using the program `dollop` from the `Phylip` package [11]. For that you need to specify an input tree in Newick format with `-t`:

```
seedVicious -i input_aligned_transcripts.fas -m input_microRNAs.fas
            -t input_tree.nwk -x 3
```

Needless to say, the input transcript file should be an alignment. In this option, microRNAs are always merged into 'seed' families (`-f`).

The output produces two trees and a number. The number ('Number of changes required') is the total number of gains/losses in the tree, as inferred by `dollop`. The first tree is a Newick tree of number of gains (+) and losses (-):

```
((transcript_A +2|-0,transcript_A +0|-1) +0|-0,transcript_A +0|-1);
```

A practical example, including how to interpret the tree, is presented in the next chapter.

A second tree includes the actual microRNA target sites gained or lost in each lineage:

```
((transcript_A |+miR-1-5p>12|+miR-2-5p>42,transcript_A ...
```

3.3.4 -x 4: Pairs of targets

In our lab we found particularly useful to find transcripts having 2 or more targets, and also measure the minimum distance between target pairs. The rationalle is that, according to a recent thermodynamic model, pairs of close target sites increase the amount of time the Ago/microRNA complex is in the 3'UTR and, therefore, the repression efficiency increases [7]. Example:

```
seedVicious -i input_transcripts.fas -m input_microRNAs.fas -x 4
```

An output table would be like this one:

```
transcript      microRNA       target_sites    min_distance
transcript_A    miR-4943-5p    3               157
transcript_B    miR-33-3p      2               618
transcript_C    miR-4961-3p    2               24
```

3.4 Web-server version

For those afraid of the command line, or just for a quick exploration of results, you may use our web-server. Please note that it can be slow, particularly with large datasets, so I strongly recommend you run the stand-alone version if possible. The web-server (Figure 3.1 is available here: `http://seedvicious.`

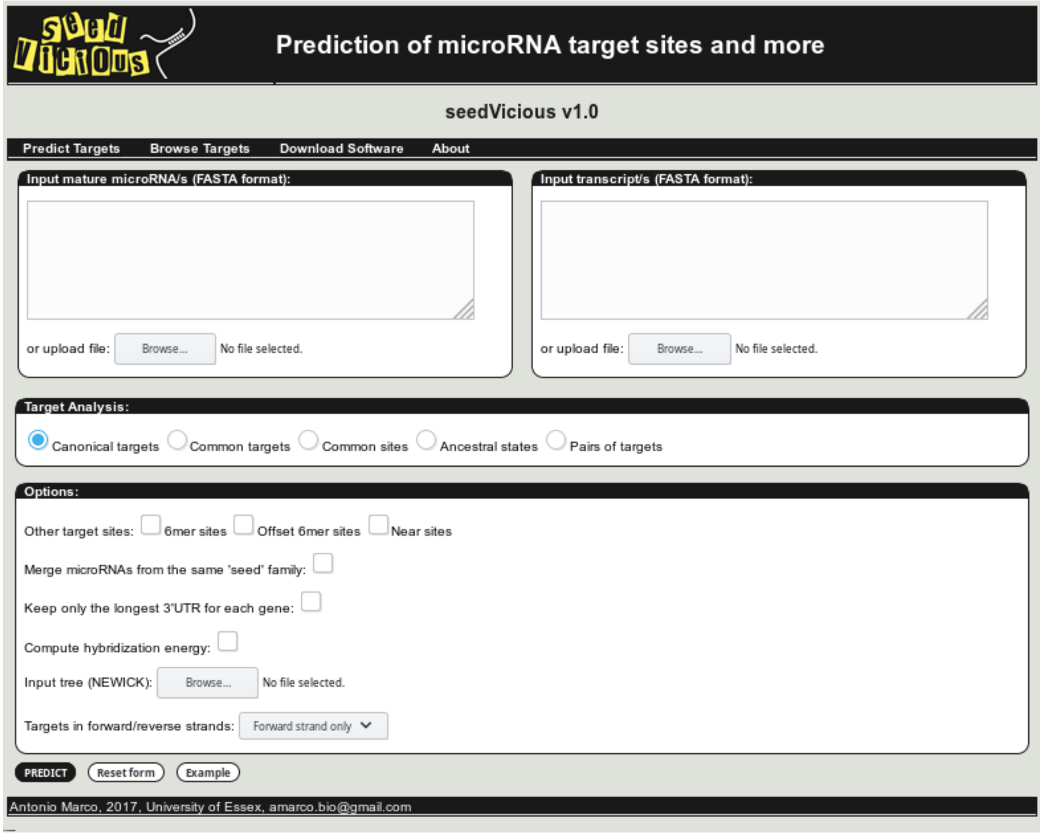

Figure 3.1: seedVicious web-server, hosted at the University of Essex

`essex.ac.uk/`, and you should click on the 'Predict Targets' tab to upload your own sequences.

The options are mostly identical to that of the stand-alone version, although the output is less convinient for high-throughput analyses.

3.5 SeedBank database of pre-computed targets

For a quick search in selected genomes (human, fly, worm, mouse, rat and chimp) you can browse a database of pre-computed targets (SeedBank) also from our web-server. Click on the 'Browse Targets' tab and fill the form for either a microRNA or a transcript. Use ENSEMBL gene names when

MicroRNA: hsa-let-7a-3p

MicroRNA family: hsa-let-7a-3p;hsa-let-7b-3p

Gene name	Gene	Transcript	Targets	Positions
INTS6	ENSG00000102786	ENST00000442263	11	436[8mer];794[7_A1];2349[7_m8];2857[7_A1];5102[7_m8];7900[7_m8];7930[7_A1];10689[
PPM1L	ENSG00000163590	ENST00000295839	11	680[7_m8];1795[7_m8];2155[7_A1];3232[7_m8];3653[7_m8];3684[8mer];7069[8mer];7681[
STX7	ENSG00000079950	ENST00000367937	10	100[8mer];1125[7_m8];5615[7_m8];6554[7_A1];7522[8mer];8214[7_m8];8736[7_m8];8886[
MPP6	ENSG00000105926	ENST00000625307	9	275[7_m8];1092[7_m8];1166[7_m8];3471[7_A1];3846[7_A1];4286[7_m8];4375[7_m8];6532
MGAT4C	ENSG00000182050	ENST00000547225	9	2787[8mer];3178[7_A1];4857[7_m8];5596[7_m8];5963[7_A1];8515[7_A1];11828[7_A1];190
SLITRK5	ENSG00000165300	ENST00000325089	8	3133[7_m8];5364[7_m8];5598[7_m8];11107[7_A1];11720[8mer];13296[7_A1];17664[7_A1];
LNPEP	ENSG00000113441	ENST00000231368	8	3996[7_m8];4359[7_m8];4380[7_A1];4677[7_A1];4887[7_A1];5235[7_m8];5339[7_A1];882
FLRT2	ENSG00000185070	ENST00000553627	7	3941[7_m8];12008[8mer];16090[7_m8];17839[7_A1];19369[7_m8];25148[7_A1];26823[7_
FUT9	ENSG00000172461	ENST00000483933	7	491[7_A1];3880[7_A1];6042[7_m8];8692[7_A1];9961[7_A1];10251[8mer];11191[7_A1]
FYTTD1	ENSG00000122068	ENST00000494309	7	1067[7_m8];1276[7_m8];1475[7_m8];1909[7_m8];2099[7_m8];3367[7_m8];4728[7_A1]
APOOL	ENSG00000155008	ENST00000613473	7	363[7_A1];389[7_A1];411[7_A1];443[7_A1];473[7_A1];503[7_A1];535[7_A1]
SLC25A16	ENSG00000122912	ENST00000609923	7	2419[7_A1];2431[7_A1];2463[7_A1];2513[7_A1];2559[7_m8];4912[7_A1];5010[7_m8]
GTF2A1	ENSG00000165417	ENST00000298173	6	484[8mer];858[7_m8];1237[7_m8];1454[7_m8];2127[7_m8];3282[7_m8]
FRS2	ENSG00000166225	ENST00000550169	6	925[7_m8];3259[7_A1];3751[8mer];3835[8mer];3914[7_m8];4113[7_m8]
PCDH9	ENSG00000184226	ENST00000544246	6	5372[7_m8];7236[8mer];10727[8mer];24016[7_m8];24028[7_A1];24175[7_m8]
KLRD1	ENSG00000134539	ENST00000381908	6	4919[7_m8];6461[7_A1];6768[8mer];7709[8mer];8871[7_A1];10663[7_m8]
SLC35A3	ENSG00000117620	ENST00000532693	6	8[7_m8];896[8mer];3722[7_m8];4202[8mer];4342[7_m8];4380[7_A1]
PEX5L	ENSG00000114757	ENST00000392649	6	193[7_m8];628[7_m8];2976[8mer];3176[7_A1];3723[7_m8];3742[7_m8]
PLAG1	ENSG00000181690	ENST00000429357	6	112[8mer];408[8mer];4346[7_A1];4959[7_m8];5067[8mer];5104[7_A1]
PUM2	ENSG00000055917	ENST00000403432	6	691[7_m8];1247[8mer];1700[7_A1];1822[8mer];2796[7_m8];2805[7_A1]
DGKH	ENSG00000102780	ENST00000498255	6	1424[7_m8];2625[7_m8];5685[7_m8];6589[7_m8];6897[7_m8];7025[8mer]
EEA1	ENSG00000102189	ENST00000549790	6	844[8mer];2592[7_A1];3091[8mer];3418[7_A1];4049[7_m8];5317[7_m8]
RICTOR	ENSG00000164327	ENST00000509567	6	1189[7_m8];1246[7_m8];1375[7_A1];4148[8mer];4214[7_A1];4289[8mer]
SLAIN2	ENSG00000109171	ENST00000510595	6	419[7_A1];1130[7_A1];1677[7_A1];2222[7_m8];2751[8mer];2890[7_m8]
GRIN2B	ENSG00000273079	ENST00000627535	6	1599[7_A1];2404[7_m8];5180[7_A1];5614[8mer];8648[7_m8];22439[7_A1]
UBE3A	ENSG00000114062	ENST00000397954	6	1171[7_m8];2559[7_m8];2920[7_A1];4894[7_m8];4907[7_m8];5338[7_m8]
GABRG1	ENSG00000163285	ENST00000295452	5	2187[7_m8];2969[7_A1];3285[7_A1];4690[7_A1];5035[8mer]
VAPA	ENSG00000101558	ENST00000577901	5	205[7_m8];2376[7_m8];2568[7_m8];4204[7_A1];5700[7_m8]
GPRIN3	ENSG00000185477	ENST00000609438	5	30[7_A1];338[8mer];7329[7_A1];8255[7_m8];9666[7_A1]
NHLRC2	ENSG00000196865	ENST00000468890	5	448[8mer];1465[7_A1];1633[7_A1];3046[8mer];7682[7_m8]

Figure 3.2: Targets in SeedBank for human microRNA hsa-let-7a-3p

possible. For instance, for human hsa-let-7a-3p an output will be as in Figure 3.2.

4

Protocols

To run this protocols, you should first install seedVicious and then navigate to the folder containing the example datasets. For instance, if you installed seedVicious in `/home/username/software/seedVicious`, you shoud you start your Bash session by typing:

```
cd /home/username/software/seedVicious/datasets
```

4.1 Detection of targets and near target between lin-4 and lin-14

lin-4 was the first described microRNA encoding gene [23], and it was found in the roundworm *C. elegans*. It's mature product, lin-4-5p, binds to the 3' UTR of the transcript produced by the gene *lin-14* [38]. Here we are going to explore this relationship.

Predict canonical target sites for lin-4-5p in lin-14 transcript, computing the hybridization energy, and with detailed output. Output file is also declared:

```
seedVicious -i cel-lin-14_3UTR.fas -m cel-lin-4-5p.fas -ev
            -o lin-4_vs_lin-14_canonical.txt
```

The output file will look like this:

```
>cel-lin-4-5p@cel-lin-14:765
MicroRNA   = cel-lin-4-5p
Transcript = cel-lin-14
Position   = 765
Type       = 8mer
Energy     = -12.35 Kcal/mol

miR  3' AGUGUGAACUCCAGAGUCCCU 5'
        ||           ||||||||
tr   5' UCUUUUAAUCCAACUCAGGGA 3'


>cel-lin-4-5p@cel-lin-14:813
MicroRNA   = cel-lin-4-5p
Transcript = cel-lin-14
Position   = 813
Type       = 8mer
Energy     = -6.70 Kcal/mol

miR  3' AGUGUGAACUCCAGAGUCCCU 5'
          |    |     ||||||||
tr   5' GAAUUUCUUCUACCUCAGGGA 3'


>cel-lin-4-5p@cel-lin-14:1044
MicroRNA   = cel-lin-4-5p
Transcript = cel-lin-14
Position   = 1044
Type       = 8mer
Energy     = -14.70 Kcal/mol

miR  3' AGUGUGAACUCCAGAGUCCCU 5'
                     ||||||||
tr   5' AACUCACAACCAACUCAGGGA 3'
```

We detected three target sites. However, there are seven putative binding sites in lin-14 for lin-4-5p [13]. Let's relax our criteria and look for marginal sites as well:

```
seedVicious -i cel-lin-14_3UTR.fas -m cel-lin-4-5p.fas -e69
            -o lin-4_vs_lin-14_marginal.txt
```

This is now the output:

```
miR      tr         pos      type     en
cel-lin-4-5p    cel-lin-14      601      off6mer  -6.70 Kcal/mol
cel-lin-4-5p    cel-lin-14      618      off6mer  -2.20 Kcal/mol
cel-lin-4-5p    cel-lin-14      765      8mer     -12.35 Kcal/mol
cel-lin-4-5p    cel-lin-14      795      off6mer  -1.47 Kcal/mol
cel-lin-4-5p    cel-lin-14      813      8mer     -6.70 Kcal/mol
cel-lin-4-5p    cel-lin-14      1044     8mer     -14.70 Kcal/mol
```

Six out of seven. Not bad. But we can do better. Actually, if multiple sites are clustered together, it may be that even near-target sites may be contributing to the repression activity of lin-4 over lin-14. Let's give a try:

```
seedVicious -i cel-lin-14_3UTR.fas -m cel-lin-4-5p.fas -n
            -o lin-4_vs_lin-14_near.txt
```

Here we go:

```
miR      tr         pos      type
cel-lin-4-5p    cel-lin-14      765      8mer
cel-lin-4-5p    cel-lin-14      813      8mer
cel-lin-4-5p    cel-lin-14      1044     8mer
cel-lin-4-5p    cel-lin-14      979      7_A1*
cel-lin-4-5p    cel-lin-14      601      8mer*
cel-lin-4-5p    cel-lin-14      618      8mer*
cel-lin-4-5p    cel-lin-14      795      8mer*
cel-lin-4-5p    cel-lin-14      934      7_A1*
```

All seven putative target sites from the literature, plus a near-target at possition 979 that has not yet been described. It may worth doing some experiments.

4.2 Gains/losses of let-7 targets in lin-14

Targets for lin-4-5p in lin-14 are highly conserved. Actually, in five species of worm studies all sites remain the same. However, let-7-5p, which also targets lin-14, has a more dynamic evolution. Let's explore it.

Predict the canonical target sites for let-7-5p in the lin-4 3'UTR alignment for five worm species, using the `-a` (alignment) tag:

```
seedVicious -i lin-14_3UTR.fas -m cel-let-7-5p.fas -a
            -o let-7_vs_lin-4_alilgnment.txt
```

Here's the output:

```
miR     tr      pos     type
cel-let-7-5p    C._japonica     445|516 7_m8
cel-let-7-5p    C._japonica     945|1169        8mer
cel-let-7-5p    C._japonica     959|1183        8mer
cel-let-7-5p    C._japonica     1539|1897       7_A1
cel-let-7-5p    C._japonica     1592|1977       7_A1
cel-let-7-5p    C._elegans      356|516 8mer
cel-let-7-5p    C._elegans      809|1169        8mer
cel-let-7-5p    C._elegans      823|1183        8mer
cel-let-7-5p    C._elegans      1222|1666       8mer
cel-let-7-5p    C._elegans      1410|1897       7_A1
cel-let-7-5p    C._briggsae     312|516 7_m8
cel-let-7-5p    C._briggsae     896|1169        8mer
cel-let-7-5p    C._briggsae     1312|1666       8mer
cel-let-7-5p    C._briggsae     1426|1906       7_A1
cel-let-7-5p    C._remanei      383|516 8mer
cel-let-7-5p    C._remanei      577|836 7_A1
cel-let-7-5p    C._remanei      830|1169        8mer
cel-let-7-5p    C._remanei      844|1183        7_A1
cel-let-7-5p    C._remanei      1227|1666       8mer
cel-let-7-5p    C._remanei      1416|1897       7_A1
cel-let-7-5p    C._brenneri     389|516 8mer
cel-let-7-5p    C._brenneri     603|836 7_A1
cel-let-7-5p    C._brenneri     843|1169        8mer
cel-let-7-5p    C._brenneri     857|1183        8mer
cel-let-7-5p    C._brenneri     1221|1666       8mer
cel-let-7-5p    C._brenneri     1402|1897       7_A1
```

At a glance, we can see that the number of targets are different, although some are conserved as the position in the alignment is the same for multiple species. We can export the data and parse it so that we can reconstruct the ancestral states with specialized software. Or, we can use the in-built sedVicious functionality to do so straigh away:

```
seedVicious -i lin-14_3UTR.fas -m cel-let-7-5p.fas
            -t lin-14_3UTR.tre -x 33 -o let-7_vs_lin-4_parsimony.txt
```

At this point I recommend you install a phylogenetic tree viewer. A simple

one is `njplot` (`http://doua.prabi.fr/software/njplot`, [34])[1].

Copy the first tree into a new file called `let-7_vs_lin-14_gains_losses.tre`:

```
((((C._briggsae +1|-3,C._remanei +0|-0) +0|-0,C._brenneri +0|-0...
```

Open the file with `njplot` and click on the 'Bootstrap values' box to see the values in the internal branches. Voilá, tree in Figure 4.1 as displayed by `njplot`.

We can see that there were 3 target sites lost in the *C. briggsae* lineage. For a more complex scenario, using exactly the same approach see [9]. For publication quality figures you may consider other phylogenetic tree editors or use a vector graphics editor such as Inkscape (`https://inkscape.org/`).

4.3 Exploring lin-4 and let-7 regulated transcripts

We've seen that lin-14 contain multiple clustered lin-4 target sites. Is this situation unique? Well, let's find out which transcripts in the worm (dataset from [15]) are have two or more lin-4-5p target sites:

```
seedVicious -i cel_3UTR.fas -m cel-lin-4-5p.fas -l -x 44
            -o lin-4_2more.txt
```

We observe that only two transcripts, lin-14 (Entrez accesion number 181337) and another transcript have at least two canonical sites. However, only lin-14 have them clustered (48 nucleotides apart):

```
transcript      microRNA        target_sites    min_distance
181337|1600     cel-lin-4-5p    3               48
181659|1377     cel-lin-4-5p    2               783
```

We can relax our constraints and report marginal sites as well:

```
seedVicious -i cel_3UTR.fas -m cel-lin-4-5p.fas -l -69 -x 44
            -o lin-4_2more_marginal.txt
```

[1]If you're running a Debian-based distro such as Ubuntu or Mint, just type `sudo apt-get install njplot`

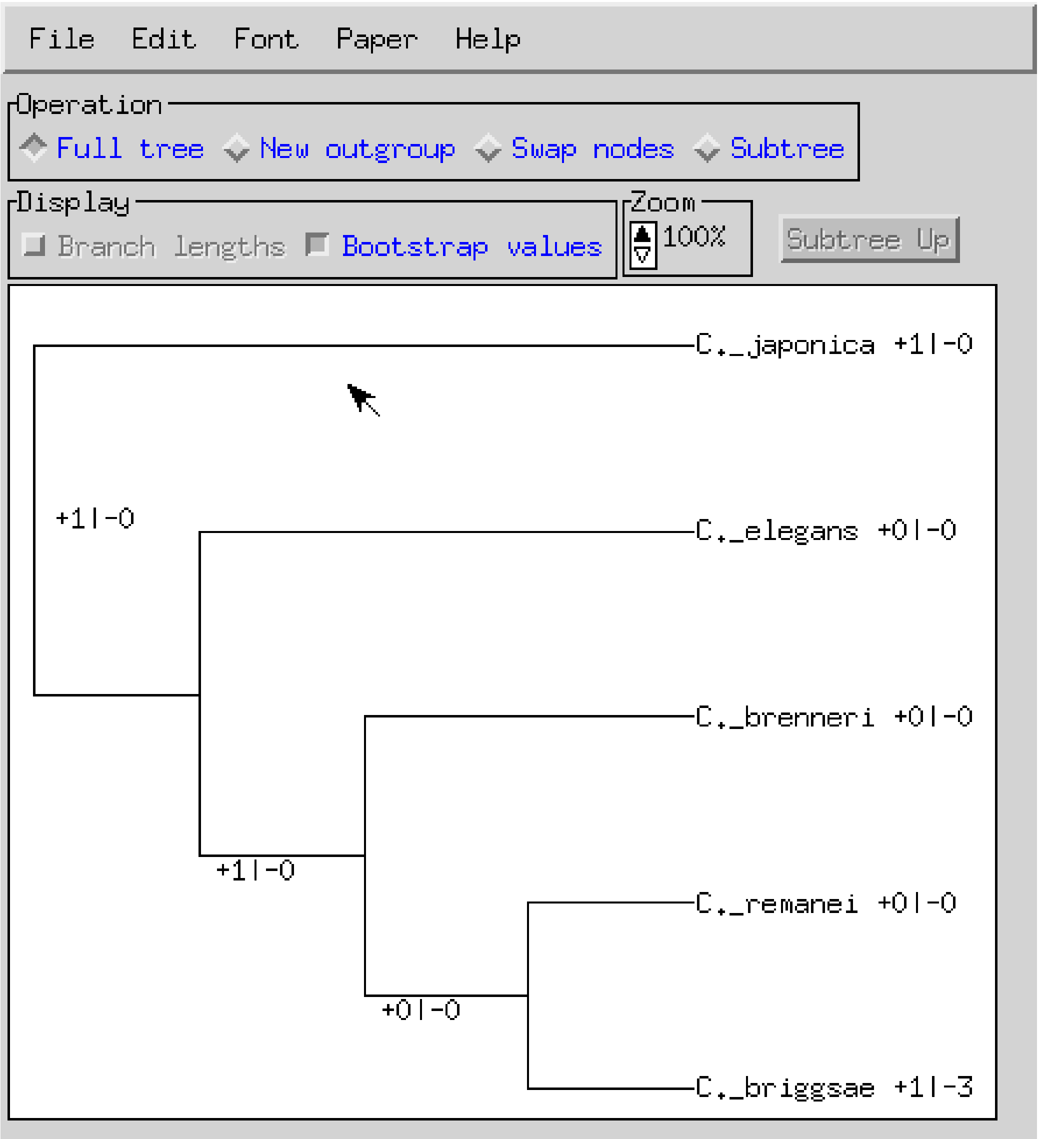

Figure 4.1: Gains and losses of let-7-5p microRNA target sites in lin-14 accross 5 worm species

With the following result:

```
transcript       microRNA         target_sites     min_distance
176548|5130      cel-lin-4-5p             3        562
181659|1377      cel-lin-4-5p             2        783
178441|882       cel-lin-4-5p             2        6
179174|448       cel-lin-4-5p             2        25
174242|258       cel-lin-4-5p             2        25
187483|1119      cel-lin-4-5p             2        609
188783|139       cel-lin-4-5p             2        6
181337|1600      cel-lin-4-5p             6        17
175351|757       cel-lin-4-5p             2        42
174907|795       cel-lin-4-5p             2        15
190787|2224      cel-lin-4-5p             2        1287
190099|1301      cel-lin-4-5p             2        42
173615|584       cel-lin-4-5p             3        74
172990|213       cel-lin-4-5p             2        22
```

In this case, another transcript (173615) corresponding to the gene encoding the hypothetical protein T05A8.3 may have three clustered lin-4-5p target sites.

We can also explore the canonical target sites for let-7-5p:

```
seedVicious -i cel_3UTR.fas -m cel-let-7-5p.fas -l -x 44
            -o let-7_2more.txt
```

Apparently, it is more common to have multiple targets for let-7 (compared to the lin-4-5p case):

```
transcript       microRNA         target_sites     min_distance
187082|630       cel-let-7-5p     3        74
186612|230       cel-let-7-5p     2        47
173873|303       cel-let-7-5p     2        24
186160|565       cel-let-7-5p     2        352
180848|1392      cel-let-7-5p     6        36
181263|1388      cel-let-7-5p     5        45
191148|820       cel-let-7-5p     2        34
181337|1600      cel-let-7-5p     5        14
183202|700       cel-let-7-5p     2        57
181181|1332      cel-let-7-5p     7        35
180867|599       cel-let-7-5p     2        238
172981|1169      cel-let-7-5p     3        247
```

4.4 Exploring alternative splicing and microRNA targets

There are 9 different isoforms known to be transcribed from the gene *crh-1* in *C. elegans.* We can find how many common targets, for a selected set of microRNAs, these isoforms have among them:

```
seedVicious -i crh-1_3UTRs.fas -m cel-miR_selected.fas -x 11
            -o crh-1_common_targets.txt
```

That looks like this:

```
transcript       transcript       common_miRs
176597|1613      176597|365       5                [cel-miR-75-5p...
176597|1613      176597|459       5                [cel-miR-75-5p...
176597|1613      176597|1555      5                [cel-miR-75-5p...
...
```

With a bit of Bash work, we can get a table of relationships among transcripts, including a weigth, whic is the number of common targets:

```
cat crh-1_common_targets.txt | grep -v -e'^#' /
        awk '{print $1 "\t" $2 "\t" $3}' > crh-1_common_targets.tab
```

This file can be use to display a graph in an appropriate piece of software. Here I opened it with Cytoscape (`http://www.cytoscape.org/`, [36]). The graphs shows, as expected, that longer isoforms are closer together as they share more common targets (Figure 4.2).

4.5 Clusters of microRNA targets

It may be interesting to explore whether certain microRNA target sites are clustered in specific transcripts. The outout of seedVicious can be easily parsed to explore this. Additionally, the package alsop provides a script to help with the task: `sv_find_clusters`. Let's try it with an example. From the `datasets` directory we will first create a file containing two microRNAs: let-7 and lin-4, and then predict their canonical targets in let-14 using seedVicious:

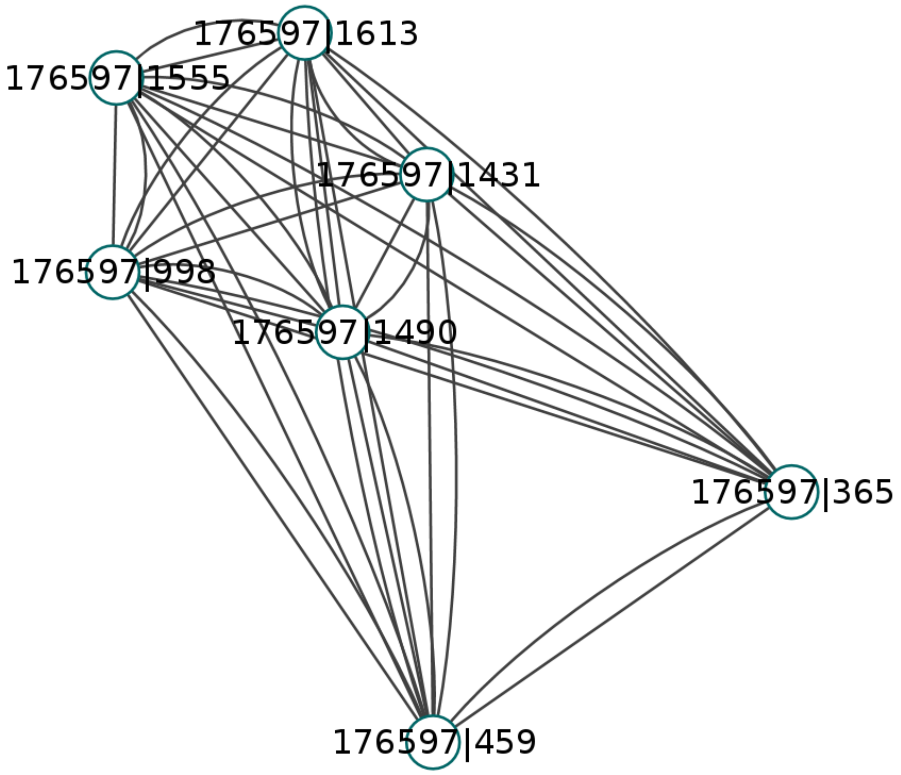

Figure 4.2: Weighted graph of transcript similarities according to common microRNA targets

```
cat cel-lin-4-5p.fas cel-let-7-5p.fas > cel_lin4_let7.fa
seedVicious -i cel-lin-14_3UTR.fas -m cel_lin4_let7.fa
        -o cel_lin4_let7_canonical.txt
```

Now, using the `sv_find_cluster` we will scan the output to find clusters of target sites less than 100 nucleotides apart, and with at least three target sites:

```
sv_find_clusters cel_lin4_let7_canonical.txt 100 3
```

And the output reports a cluster of three targets sites around position 810 of the lin-14 3'UTR:

```
cel-lin-14      cel-let-7-5p[809]/cel-lin-4-5p[813]/cel-let-7-5p[823]
```

These are but a few examples to explore some of the functions of seedVicious. The applications are endless. If you'd like to see a specific type of analysis using seedVicious, don't hesitate to contact me.

5

Frequently Asqued Questions

This is an early version of the User Guide. Therefore, no Frequently Asqued Questions, as such, exist. I will update this section with the most common questions and how to fix potential errors/bugs.

For any suggestion or question write me at `amarco.bio@gmail.com`.

Citing seedVicious

If you use either the web-server or the stand-alone version of seedVicious, please, cite this paper:

Marco A. (2017) SeedVicious: analysis of microRNA target and near-target sites. *bioRxiv* doi:10.1101/124529

Bibliography

[1] Vikram Agarwal, George W. Bell, Jin-Wu Nam, and David P. Bartel. Predicting effective microRNA target sites in mammalian mRNAs. *eLife*, 4, 2015.

[2] Panagiotis Alexiou, Manolis Maragkakis, Giorgos L Papadopoulos, Martin Reczko, and Artemis G Hatzigeorgiou. Lost in translation: an assessment and perspective for computational microRNA target identification. *Bioinformatics (Oxford, England)*, September 2009.

[3] Michael J Axtell, Jakub O Westholm, and Eric C Lai. Vive la diffÃ©rence: biogenesis and evolution of microRNAs in plants and animals. *Genome Biology*, 12(4):221, April 2011.

[4] David P Bartel. MicroRNAs: genomics, biogenesis, mechanism, and function. *Cell*, 116(2):281–297, January 2004.

[5] David P. Bartel. MicroRNAs: Target Recognition and Regulatory Functions. *Cell*, 136(2):215–233, January 2009.

[6] Julius Brennecke, Alexander Stark, Robert B Russell, and Stephen M Cohen. Principles of microRNA-target recognition. *PLoS Biology*, 3(3):e85, March 2005.

[7] Stanley D. Chandradoss, Nicole T. Schirle, Malwina Szczepaniak, Ian J. MacRae, and Chirlmin Joo. A Dynamic Search Process Underlies MicroRNA Targeting. *Cell*, 162(1):96–107, July 2015.

[8] K. Chen and N. Rajewsky. Natural selection on human microRNA binding sites inferred from SNP data. *Nat. Genet.*, 38(12):1452–1456, Dec 2006.

[9] Bryan D. Clifton, Pablo Librado, Shu-Dan Yeh, Edwin S. Solares, Daphne A. Real, Suvini U. Jayasekera, Wanting Zhang, Mijuan Shi, Ronni V. Park, Robert D. Magie, Hsiu-Ching Ma, Xiao-Qin Xia, Antonio Marco, Julio Rozas, and JosÃ© M. Ranz. Rapid Functional and Sequence Differentiation of a Tandemly Repeated Species-Specific Multigene Family in Drosophila. *Molecular Biology and Evolution*, 34(1):51–65, January 2017.

[10] Anton J Enright, Bino John, Ulrike Gaul, Thomas Tuschl, Chris Sander, and Debora S Marks. MicroRNA targets in Drosophila. *Genome Biology*, 5(1):R1, 2003.

[11] J Felsenstein. *PHYLIP (Phylogeny Inference Package) version 3.6.* Distributed by the author. Department of Genome Sciences, University of Washington, Seattle., 2005.

[12] Andrew Grimson, Kyle Kai-How Farh, Wendy K Johnston, Philip Garrett-Engele, Lee P Lim, and David P Bartel. MicroRNA targeting specificity in mammals: determinants beyond seed pairing. *Molecular Cell*, 27(1):91–105, July 2007.

[13] Lin He and Gregory J Hannon. MicroRNAs: small RNAs with a big role in gene regulation. *Nature Reviews. Genetics*, 5(7):522–531, July 2004.

[14] Ivo L Hofacker. RNA secondary structure analysis using the Vienna RNA package. *Current Protocols in Bioinformatics*, Chapter 12:Unit12.2, June 2009.

[15] Calvin H. Jan, Robin C. Friedman, J. Graham Ruby, and David P. Bartel. Formation, regulation and evolution of Caenorhabditis elegans 3[prime]UTRs. *Nature*, advance online publication, November 2010.

[16] Ana Kozomara and Sam Griffiths-Jones. miRBase: annotating high confidence microRNAs using deep sequencing data. *Nucleic acids research*, 42(Database issue):D68–73, January 2014.

[17] Ana Kozomara, Suzanne Hunt, Maria Ninova, Sam Griffiths-Jones, and Matthew Ronshaugen. Target Repression Induced by Endogenous microRNAs: Large Differences, Small Effects. *PloS One*, 9(8):e104286, 2014.

[18] Jan Kruger and Marc Rehmsmeier. RNAhybrid: microRNA target prediction easy, fast and flexible. *Nucl. Acids Res.*, 34(suppl_2):W451–454, July 2006.

[19] M. Lagos-Quintana, R. Rauhut, W. Lendeckel, and T. Tuschl. Identification of novel genes coding for small expressed RNAs. *Science*, 294(5543):853–858, Oct 2001.

[20] Eric C Lai. Micro RNAs are complementary to 3' UTR sequence motifs that mediate negative post-transcriptional regulation. *Nature Genetics*, 30(4):363–364, April 2002.

[21] N. C. Lau, L. P. Lim, E. G. Weinstein, and D. P. Bartel. An abundant class of tiny RNAs with probable regulatory roles in Caenorhabditis elegans. *Science*, 294(5543):858–862, Oct 2001.

[22] R. C. Lee and V. Ambros. An extensive class of small RNAs in Caenorhabditis elegans. *Science*, 294(5543):862–864, Oct 2001.

[23] R C Lee, R L Feinbaum, and V Ambros. The C. elegans heterochronic gene lin-4 encodes small RNAs with antisense complementarity to lin-14. *Cell*, 75(5):843–854, December 1993.

[24] Benjamin P Lewis, Christopher B Burge, and David P Bartel. Conserved seed pairing, often flanked by adenosines, indicates that thousands of human genes are microRNA targets. *Cell*, 120(1):15–20, January 2005.

[25] Benjamin P Lewis, I-hung Shih, Matthew W Jones-Rhoades, David P Bartel, and Christopher B Burge. Prediction of mammalian microRNA targets. *Cell*, 115(7):787–798, December 2003.

[26] Antonio Marco. Selection Against Maternal microRNA Target Sites in Maternal Transcripts. *G3: Genes|Genomes|Genetics*, 5(10):2199–2207, October 2015.

[27] Antonio Marco. seedVicious: a versatile microRNA target site prediction tool with evolutionary applications. *In_Preparation*, 2017.

[28] Antonio Marco, Katarzyna Hooks, and Sam Griffiths-Jones. Evolution and function of the extended miR-2 microRNA family. *RNA Biology*, 9(3):242–248, March 2012.

[29] Antonio Marco, Jamie I. MacPherson, Matthew Ronshaugen, and Sam Griffiths-Jones. MicroRNAs from the same precursor have different targeting properties. *Silence*, 3(1):8, September 2012.

[30] Antonio Marco, Maria Ninova, and Sam Griffiths-Jones. Multiple products from microRNA transcripts. *Biochemical Society transactions*, 41(4):850–854, August 2013.

[31] Maria Ninova, Matthew Ronshaugen, and Sam Griffiths-Jones. MicroRNA evolution, expression and function during short germband development in Tribolium castaneum. *Genome Research*, October 2015.

[32] M. D. Paraskevopoulou, G. Georgakilas, N. Kostoulas, I. S. Vlachos, T. Vergoulis, M. Reczko, C. Filippidis, T. Dalamagas, and A. G. Hatzigeorgiou. DIANA-microT web server v5.0: service integration into miRNA functional analysis workflows. *Nucleic Acids Res.*, 41(Web Server issue):W169–173, Jul 2013.

[33] A E Pasquinelli, B J Reinhart, F Slack, M Q Martindale, M I Kuroda, B Maller, D C Hayward, E E Ball, B Degnan, P MÃ$\frac{1}{4}$ller, J Spring, A Srinivasan, M Fishman, J Finnerty, J Corbo, M Levine, P Leahy, E Davidson, and G Ruvkun. Conservation of the sequence and temporal expression of let-7 heterochronic regulatory RNA. *Nature*, 408(6808):86–89, November 2000.

[34] G. Perriere and M. Gouy. WWW-query: an on-line retrieval system for biological sequence banks. *Biochimie*, 78(5):364–369, 1996.

[35] B J Reinhart, F J Slack, M Basson, A E Pasquinelli, J C Bettinger, A E Rougvie, H R Horvitz, and G Ruvkun. The 21-nucleotide let-7 RNA regulates developmental timing in Caenorhabditis elegans. *Nature*, 403(6772):901–906, February 2000.

[36] P. Shannon, A. Markiel, O. Ozier, N. S. Baliga, J. T. Wang, D. Ramage, N. Amin, B. Schwikowski, and T. Ideker. Cytoscape: a software environment for integrated models of biomolecular interaction networks. *Genome Res.*, 13(11):2498–2504, Nov 2003.

[37] Alexander Stark, Julius Brennecke, Robert B Russell, and Stephen M Cohen. Identification of Drosophila MicroRNA targets. *PLoS Biology*, 1(3):E60, December 2003.

[38] B Wightman, I Ha, and G Ruvkun. Posttranscriptional regulation of the heterochronic gene lin-14 by lin-4 mediates temporal pattern formation in C. elegans. *Cell*, 75(5):855–862, December 1993.
